# A Novel Ex Vivo System Using 3D Polymer Scaffold to Culture Circulating Tumor Cells from Breast Cancer Patients Exhibits Dynamic E-M Phenotypes

**DOI:** 10.3390/jcm8091473

**Published:** 2019-09-16

**Authors:** Tamasa De, Shina Goyal, Gowri Balachander, Kaushik Chatterjee, Prashant Kumar, Govind Babu K., Annapoorni Rangarajan

**Affiliations:** 1 Department of Molecular Reproduction, Development and Genetics, Indian Institute of Science, Bangalore 560012, India; tamasade@iisc.ac.in; 2 Department of Medical Oncology, Kidwai Memorial Institute of Oncology, Bangalore 560029, India; shina.goyal@gmail.com; 3 Center for Biosystems Science and Engineering, Indian Institute of Science, Bangalore 560012, India; phsgmb@nus.edu.sg (G.B.); kchatterjee@iisc.ac.in (K.C.); 4 Department of Physiology, Yong Loo Lin School of Medicine, National University Health System, MD9-04-11, 2 Medical Drive, Singapore 117593, Singapore; 5 Department of Materials Engineering, Indian Institute of Science, Bangalore 560066, India; 6 Institute of Bioinformatics, International Technology Park, Bangalore 560066, India; 7 Manipal Academy of Higher Education (MAHE), Manipal 576104, Karnataka, India

**Keywords:** circulating tumor cells (CTCs), breast cancer, 3D culture, epithelial-mesenchymal heterogeneity

## Abstract

The majority of the cancer-associated deaths is due to metastasis—the spread of tumors to other organs. Circulating tumor cells (CTCs), which are shed from the primary tumor into the circulation, serve as precursors of metastasis. CTCs have now gained much attention as a new prognostic and diagnostic marker, as well as a screening tool for patients with metastatic disease. However, very little is known about the biology of CTCs in cancer metastasis. An increased understanding of CTC biology, their heterogeneity, and interaction with other cells can help towards a better understanding of the metastatic process, as well as identify novel drug targets. Here we present a novel ex vivo 3D system for culturing CTCs from breast cancer patient blood samples using porous poly(ε-caprolactone) (PCL) scaffolds. As a proof of principle study, we show that ex vivo culture of 12/16 (75%) advanced stage breast cancer patient blood samples were enriched for CTCs identified as CK+ (cytokeratin positive) and CD45− (CD45 negative) cells. The deposition of extracellular matrix proteins on the PCL scaffolds permitted cellular attachment to these scaffolds. Detection of Ki-67 and bromodeoxyuridine (BrdU) positive cells revealed proliferating cell population in the 3D scaffolds. The CTCs cultured without prior enrichment exhibited dynamic differences in epithelial (E) and mesenchymal (M) composition. Thus, our 3D PCL scaffold system offers a physiologically relevant model to be used for studying CTC biology as well as for individualized testing of drug susceptibility. Further studies are warranted for longitudinal monitoring of epithelial–mesenchymal transition (EMT) in CTCs for clinical association.

## 1. Introduction 

Most of the cancer-related mortality is caused due to metastasis—the spread of cancer to secondary vital organs [1,2]. This is a complex phenomenon involving dissemination of cells from the primary site, intravasation into the circulatory system followed by extravasation, and finally successful colonization in secondary tumor sites such as liver, lung, bone and brain [1,2]. Cancer that is diagnosed at the primary site is relatively easier to manage compared to those with metastatic lesions. Circulating tumor cells (CTCs) derived from either the primary or metastatic tumors serve as precursors of metastasis [3]. To begin to understand the biology of CTCs and their role in the metastatic process, it is important to culture CTCs in a suitable microenvironment that recapitulates their physiological features.

CTCs represent an extraordinarily rare population in the milieu of billions of blood cells, and hence, their identification and isolation pose critical impediments to their characterization [4]. In the past decade or so, several technologies have come up to isolate and detect CTCs. Broadly CTC detection is done by two methods, one involving pre-enrichment with markers, and the other without enrichment, which is also known as direct detection of CTCs. The reported technologies for direct detection include (1) line-confocal microscopy and (2) surface-enhanced Raman scattering nanoparticles (SERS) [5,6]. On the other hand, marker-based pre-enrichment methods include several techniques, for example, Cell Search™, Dynabeads^®^ CD45, EPISPOT (EPithelial ImmunoSPOT), ClearCell^®^ FX1 System, Herringbone CTC-Chip, CTC-iChip, DEPArray™ System, etc. [4,7,8,9]. However, pre-enrichment results in the loss of CTCs that do not express the chosen markers. Further, several detection techniques involve cell-fixation which does not allow subsequent CTC expansion for biological characterization including stemness, drug resistance, etc. In addition, most of these methods detect only low numbers of CTCs (<20%) [10]. Hence, there is an unmet need to establish a robust method with improved efficiency for CTC enrichment to enable a better understanding of their biology.

Conventional two-dimensional (2D) culture system suffers from major limitations in terms of altered cellular morphology, motility, polarity and other functional aspects. When grown on a 2D substrate, cells lose their in vivo morphology and importantly their cell–cell and cell–matrix interactions. Further, stiffness of materials typically used for 2D cell cultures, such as tissue culture polystyrene (TCPS) and glass, are several orders of magnitude (GigaPascals) higher than the stiffness of human tissues (kiloPascals) [11]. In addition, studies have also demonstrated altered signaling in cancer cells when cultured in 2D platforms [12]. Therefore, we sought to develop a three-dimensional (3D) culture system to enrich and expand the rare population of CTCs.

Three-dimensional culture systems have been widely explored to study breast cancer over the past three decades. A variety of 3D gel-based matrices like collagen, Matrigel, laminin-rich extracellular matrix (lrECM), fibrin, etc., have been routinely used to mimic cell-basement membrane interactions. However, the abundance of ECM already present in these matrices reduces the secretion of native ECM molecules by cancer cells [12,13]. To overcome these limitations, we recently developed a 3D porous scaffold synthesized using a synthetic biomaterial, poly(ε-caprolactone) (PCL) that better mimics the architecture and stiffness of breast tumors, and enables deposition of native ECM [13]. Our 3D culture system showed improved cell–cell and cell–matrix interactions. Furthermore, global microarray analysis revealed that cancer cells cultured in this 3D scaffold are able to maintain increased stemness and epithelial–mesenchymal transition (EMT) properties, and show closer association to in vivo tumor growth than conventional 2D cultures on TCPS [13]. Thus, this 3D porous PCL scaffold system offers a superior model system to mimic the native tumor microenvironment.

In this study, we have exploited the 3D porous PCL scaffold to enrich CTCs derived from breast cancer patient blood samples without any prior enrichment. We have previously shown that culture of patient blood cells under hypoxic conditions for 14 days in laser ablated microwells helps in the enrichment of CTC subpopulation from RBC lysed nucleated cell fraction [14] Using various lineage-specific markers, we further showed gradual depletion of other blood cell lineages with time [14] More recently, we used a similar strategy to expand CTCs in agar-microwells [15]. However, being inert, agar does not allow deposition of extracellular matrix. Therefore, here, we established a 3D PCL scaffold-based method, which better mimics the native cellular in vivo environment and allows ECM deposition, for culturing CTCs from RBC-depleted nucleated cell pellets of advanced breast cancer patient samples under hypoxic conditions. We detected CK-positive and CD45-negative CTCs in 12/16 patient samples using this culture method. We detected the deposition of thread-like and sheet-like ECM on the scaffold providing a substratum for cells to adhere and proliferate. Our study shows intra-patient and inter-patient heterogeneity with respect to the epithelial (E) and mesenchymal (M) characteristics suggesting a dynamic EMT spectrum in CTCs. Thus, we established a unique model system by creating in vivo-like functionality to culture CTCs from whole blood samples without prior enrichment. Our model may provide a versatile system for studying CTC biology as well as in the use of a number of downstream exciting applications for the clinical utility of CTCs.

## 2. Materials and Methods

### 2.1. Patients Sample, Blood Collection and Processing 

Blood samples were collected from 16 chemo-naïve metastatic breast cancer patients. This study was approved by the institutional review board at Indian Institute of Science (IISc) and at the Kidwai Memorial Institute of Oncology (KMIO/MEC/017/23.March.2017, KMIO/MEC/018/23.March.2017, KMIO/MEC/011.November.2016). All patients gave their informed consent for the completion of the study. Among 16 patients (median age of breast cancer patients = 50.5), 31 patients were ER/PR positive (81.25%), 2 patients were triple negative (12.5%), and 8 patients were HER-2 positive (50%). Out of all these 16 patients with breast cancer, 1 patient had only brain metastasis (6.25%), 14 patients had liver, lung and bone metastasis (87.5%), and 3 patient had contralateral axillary and lymph node metastasis along with lung/liver/bone metastasis (Supplementary Table S1). Clinicopathological information was recorded for each patient. Blood sample (~10 mL) was collected at a single draw in chemo-naïve conditions. All the samples were collected in sterile EDTA-coated vacutainer tubes (BD) and maintained at 4 °C until processing. 

Blood samples were processed within 3–6 h of withdrawal to avoid blood clotting and to maintain cell viability. In order to isolate nucleated cells, plasma and blood cells were separated from whole blood by centrifuging at 1200 rpm for 10 min. Blood cells were then treated with chilled red blood cell (RBC) lysis buffer (154 mM NH_4_Cl, 10 mM KHCO_3_, 0.1 mM EDTA) at a ratio of 1:5 with a gentle-mixing followed by incubation at room temperature (25 °C) for 15 min. Next, the whole content was centrifuged at 1200 rpm for 10 min at room temperature to remove lysed RBC fragments. The leftover, largely nucleated cells following RBS lysis were resuspended in fresh Dulbecco’s modified Eagle’s medium (DMEM; Sigma-Aldrich, Saint Louis, MO, USA) supplemented with 10% fetal bovine serum (FBS, Gibco, Invitrogen, Carlsbad, CA, USA) containing antibiotics streptomycin sulphate and benzylpenicillin at final concentrations of 100 μg/mL and 100 U/mL, respectively. Each processed sample was split into multiple wells of a 96-well plate (~10 mL RBC-lysed blood was distributed into 5 or 6 wells).

### 2.2. Preparation of 3D Scaffold System 

Fabrication of 3D PCL porous scaffolds is mentioned in detail elsewhere [13]. Briefly, sodium chloride (Fisher scientific, Hampton, Hampshire, USA) crystals of a defined size range of 250–425 μm were used as the porogen. PCL (average molecular weight M_n_ = 80,000 g/mol; Sigma) was dissolved in chloroform and added on to the salt bed in 96 polypropylene plate. Finally, it was vacuum dried, and the salt was leached in dH_2_O for three consecutive days with daily change of water to completely leach the salt. Subsequently, the morphology, pore size and pore interconnectivity were confirmed by scanning electron microscopy (SEM). Finally, scaffolds were made sterile using 70% ethanol wash followed by UV exposure for 30 min before using for tissue culture. 

### 2.3. Culture of CTCs in 3D PCL Scaffolds

The RBC-lysed nucleated cells were seeded in scaffolds and maintained at 37 °C in 5% (*v*/*v*) CO_2_ and 1.1% O_2_ under humidified conditions. Each processed sample was split into multiple wells of a 96-well plate (~10 mL RBC-lysed blood was distributed into 5 or 6 wells). The culture medium (DMEM + 10% FBS) was changed after 72 h the first time after seeding, followed by every alternative day up to 14 days.

### 2.4. Immunophenotyping of Cells

Cells and scaffolds were fixed with 3.7% formaldehyde for 15 min and permeabilized with 0.2% Triton X-100 for maximum 10 min followed by three washes with PBST (0.05% Tween 20 in 1× PBS) each for 5 min. Blocking was done with blocking buffer (0.2% fish skin gelatin: FSG, 0.01% Tween 20, 0.2% sodium azide: NaN_3_) for 45 min. Primary antibodies were used at 1:200 dilutions for overnight at 4 °C. Excess antibodies were removed by giving three washes with PBST each for 5 min. Secondary antibodies were used at 1:200 dilutions and incubated at dark for 2 h at room temperature. In some studies, Phalloidin conjugated with Alexa Fluor™ 488 and Alexa Fluor™ 546 were used for 2 h in dark. Counterstaining for nucleus with Hoechst 33342 was done by incubating for 5 min in dark. All the samples were imaged using epifluorescence (Olympus IX71) or confocal laser (Olympus FV10i/Olympus FV3000/Leica SP8) microscope. Image processing was performed by ImageJ software.

### 2.5. Study of Cell Morphology by Scanning Electron Microscopy (SEM)

RBC-depleted nucleated cells from patient blood sample cultured in scaffolds were fixed with 2.5% glutaraldehyde for 12 h at 4 °C. The cell-laden scaffolds were then dehydrated in a gradient of ethanol, 30, 50, 70, 90, and 100%, each for a period of 10 min. The samples were completely air-dried and gold-coated by means of sputtering apparatus before observation to avoid charging under the electron beam. The samples were analyzed using SEM microscopy (JEOL SEM, Peabody, MA, USA).

### 2.6. Bromodeoxyuridine (BrdU) Assay for Cell Proliferation in 3D PCL Scaffolds

#### 2.6.1. Immunofluorescence-Based BrdU Assay

For immunostaining, RBC-depleted nucleated cells cultured in 3D PCL scaffolds (at day 7, day 14) were permeabilized with 0.2% Triton X-100 on ice for 90 sec followed by fixation with 3.7% formaldehyde at RT. Cells were denatured with denaturing solution (2N HCl, 0.5% Triton X-100) for 30 min at RT, followed by blocking (0.5% BSA, 0.5% Triton X-100) for 30 min at RT. Primary anti-BrdU was used at 1:1000 dilution for 2 h at RT. Excess antibodies were removed by washing thrice with PBST. Secondary antibody was used at 1:200 dilution for 1 h at RT in dark, followed by three washes with PBST. Finally, counterstaining for nucleus was done with Sytox green (Invitrogen, Carlsbad, CA, USA) for 5 min at RT in dark. Imaging was done in Leica SP8 confocal laser microscope. Images were processed with ImageJ software.

#### 2.6.2. Colorimetry-Based BrdU Assay

RBC-depleted nucleated cells from blood samples of breast cancer patients were cultured in 3D PCL scaffolds for different day points (day 3, day 7, day 14) under hypoxic condition. MDA-MB-231 cells were cultured under a similar condition which was taken as control. Further, similar cells were cultured in 2D culture system for 24 h under normoxia which was taken as the 2D control. On the particular day point, BrdU label (1:2000 dilution in tissue culture media) was added and left for 48 h for incubation inside the incubator. Scaffolds containing cells were then fixed and denatured using fixative or denaturing solution (Calbiochem QIA58) by incubating for 30 min at room temperature (RT). Primary anti-BrdU antibody was used at 1:100 dilutions for 1 h at RT. Excess antibody was removed by using 1× wash buffer, provided with the kit. HRP conjugated secondary antibody was used at 1:1000 dilution for 30 min at RT followed by three washes with wash buffer. Wells were flooded with ddH_2_O. Next, the substrate (TMB-tetramethylbenzidine) was added in the well and was incubated in dark for 15 min at 37 °C which resulted in a change of color of the solution to blue. Finally, stop solution was added which turns the solution yellow colored. Absorbance was measured at dual wavelengths, 450 nm and 595 nm, within 30 min of adding stop solution.

## 3. Results

### 3.1. Culture of Breast Cancer Patient-Derived Cells in 3D PCL Scaffolds 

**Figure 1:**
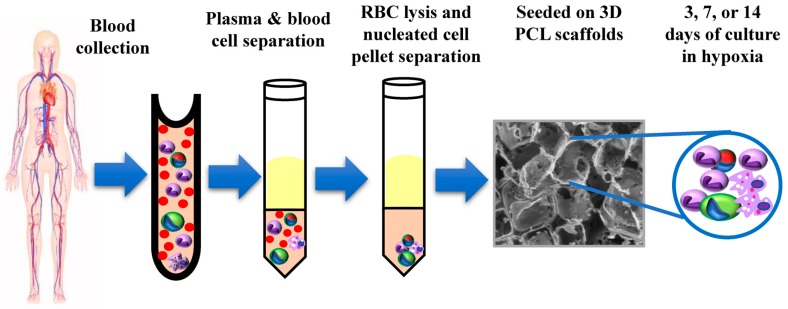
Workflow of culturing breast cancer patient blood-derived cells in 3D poly(ε-caprolactone) (PCL) scaffolds. Schematic showing the culture method of breast cancer patient blood samples for culturing circulating tumor cells (CTCs).

**Figure 2:**
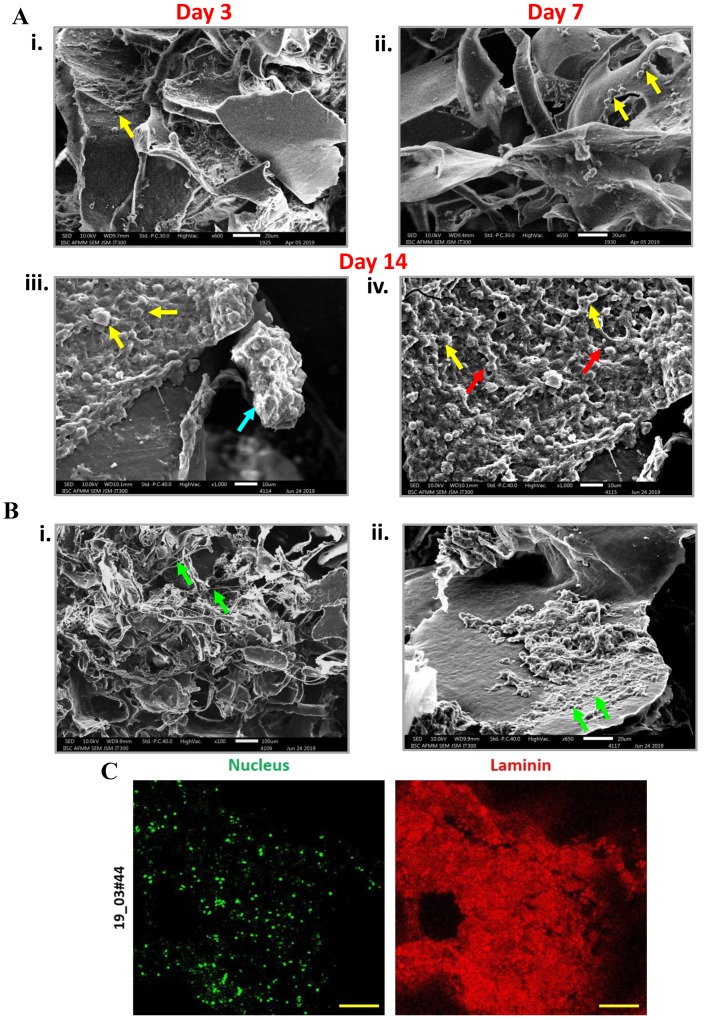
Detection of cell clusters and extracellular matrix (ECM) deposition in 3D PCL scaffolds. (**Ai–iv**) SEM micrographs showed the presence of increasing numbers of cells in day 3, day 7 and day 14 cultures of red blood cell (RBC)-depleted nucleated cell pellet of patient samples cultured in 3D PCL scaffold (yellow arrows). Formation of clusters (**iii**) (blue arrow) and the presence of intercellular contacts (**iv**) (red arrows) was detected after 14 days of culture. Scale bar represents 20 μm. Images are representative of 6 patient samples. (**B**) Scanning electron microscopy analysis revealed the deposition of thread-like (**i**) and sheet-like (**ii**) ECM (green arrows). Images are representative of 4 patient samples. (**C**) Cells were immunostained for ECM protein Laminin (red); nucleus counterstained with Hoechst 33342 (pseudocoloured green). Imaging was performed using confocal microscope and maximum intensity projections are shown. Scale bar represents 100 μm. Minus primary antibody served as negative control and did not show staining; data not shown.

In an attempt to establish CTC culture in 3D porous PCL scaffolds, blood samples were collected from 16 chemo-naïve breast cancer patients clinically diagnosed with brain, liver or lung metastases (Supplementary Table S1). Three-dimensional PCL scaffolds were fabricated as per the standardized protocol [13] (Supplementary Figure S1A). Following the separation of plasma from blood cells and RBC lysis, the nucleated cell pellet was resuspended in media and seeded in 3D PCL scaffolds (schematic in Figure 1A). After 14 days, the scaffold culture was stained and imaged. Fluorescence micrographs showed positivity for nucleus and F-actin, revealing the presence of nucleated cells (Supplementary Figure S1B). These observations were corroborated with scanning electron microscopy with SEM micrographs showing the presence of fewer cells after 3 days and 7 days of culture (Figure 2Ai,ii), which gradually increased by 14 days of culture (Figure 2Aiii,iv). We also observed the presence of cells attached to scaffolds by day 7 of culture (yellow arrow, Figure 2Aii) and the presence of cell clusters by day 14 of culture in 3D PCL scaffolds (blue arrow, Figure 2Aiii) as well as intercellular connections (red arrow, Figure 2Aiv). Interestingly, we noted the deposition of thread-like (Figure 2Bi) and sheet-like (Figure 2Bii) extracellular matrix (ECM) in the scaffold culture. Immunostaining for the ECM protein laminin confirmed ECM deposition by cells on the PCL scaffold (Figure 2C). These observations suggested the significant capture and expansion of nucleated cells from the blood in 3D PCL scaffolds.

### 3.2. Detection of CK-Positive and CD45-Negative Cells in 3D Scaffold Cultures

**Figure 3:**
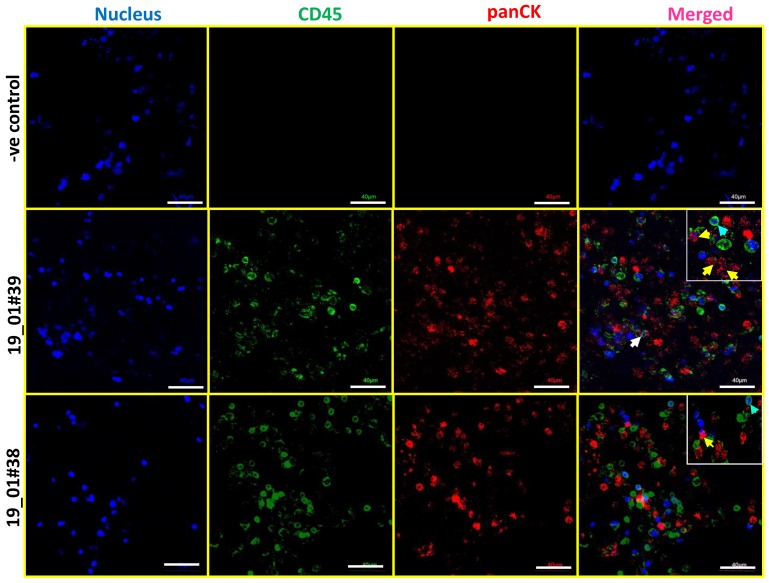
Identification of CK-positive and CD45-negative CTCs in breast cancer patient blood samples cultured in 3D PCL scaffold. Immunostaining shows the presence of panCK+/CD45− CTCs in day 14 cultures of patient blood samples cultured in 3D PCL scaffolds. Top panel shows negative control (minus primary antibody). Middle and bottom panels show immunostaining done on two different patient samples. Yellow arrows indicate panCK+ and CD45 negative CTCs while blue arrows indicate CD45 positive leucocyte lineage cells. White arrow shows CK+ cells surrounded by CD45+ cells. Imaging was performed using confocal microscope, and maximum intensity projections are shown. Inset shows higher magnification. Scale bar represents 40 μm. Images are representative of 7 independent patient samples.

CTCs immunocaptured by epithelial cell adhesion molecule (EpCAM) antibodies in devices including FDA-approved CellSearch are often identified as cytokeratin (CK)-positive and CD45-negative cells [16,17,18]. Therefore, we sought to identify CK+/CD45− cells in 3D PCL scaffolds by dual staining. We undertook immunostaining for panCK and CD45 concomitant with a nuclear counterstain (Hoechst 33342). Confocal microscopy confirmed the presence of panCK-positive and CD45-negative cells derived from blood samples of breast cancer patients (Figure 3). Thus, the detection of CK-positive and CD45-negative cells confirmed the presence of breast cancer patient-derived CTCs in the 3D PCL scaffolds. In addition, we also observed CK+ cells surrounded by CD45+ cell.

### 3.3. Cell Proliferation in 3D PCL Scaffolds 

**Figure 4:**
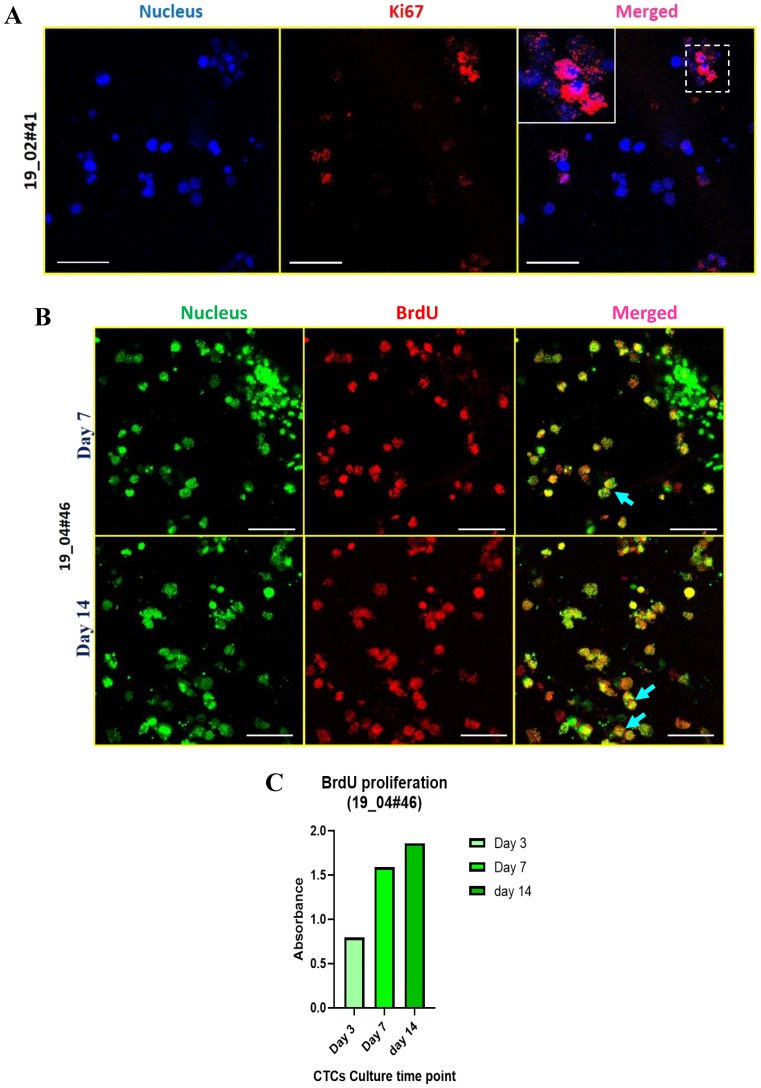
Active cell proliferation in breast cancer patient-derived cells cultured in 3D PCL scaffolds. (**A**) Fluorescence images of Ki-67 positive patient blood cells harvested after 14 days of culture in 3D PCL scaffolds. Cells were stained for nucleus (Hoechst 33342, blue), Ki-67 (red). Images are representative of 2 independent patient samples. (**B**) Patient-derived cells were exposed to a single pulse of 50 μM BrdU on day 7 and day 14 of culture and harvested after 48 h for immunostaining for BrdU incorporation. Fluorescence images show BrdU-positive cells (red), stained for nucleus (Sytox green); blue arrows show the merge. Imaging was performed using confocal microscope and maximum intensity projections are shown. Scale bar represents 40 μm (**A**,**B**). (**C**) Graph shows BrdU incorporation in patient-derived cells over time (day 3, day 7, day 14) in culture as measured by colorimetry. Images are representative of 2 independent patient samples.

After establishing a novel 3D system to culture CTCs from patient-blood derived nucleated cells, we tested whether this culture system facilitates cell proliferation. After testing the expression of Ki-67, a proliferative marker, in actively dividing MDA-MB-231 cells as a positive control (Supplementary Figure S2A,B) we checked the expression of Ki-67 in the RBC-depleted nucleated cells cultured in the PCL scaffolds for 14 days. Fluorescence images revealed distinct nuclear localization of Ki-67 (Figure 4A), suggesting that these cells are actively proliferating in 3D PCL scaffolds. This observation was confirmed in 2 independent breast cancer patient samples cultured in the scaffolds.

To further confirm active cell proliferation, we performed BrdU-based cell proliferation assay. After exposing day 7 and day 14 cultures to BrdU for 48 h, we did immunostaining using anti-BrdU-specific antibodies. We detected around 10 out of 30 cells (per field) as BrdU-positive cells (blue arrows) by 7 days of culture, whereas approximately 20 out of 30 cells were BrdU-positive by 14 days of culture (Figure 4B). Further, we performed a colorimetry-based assay to validate the fluorescence-based observation of BrdU incorporation. We exposed cells cultured in the 3D scaffold to BrdU label for 48 h at different time points, including day 3, day 7 and day 14. The graph revealed a gradual increase in BrdU incorporation (based on relative absorbance) (Figure 4C). Together, these data revealed active cell proliferation in 3D PCL scaffolds. 

### 3.4. Epithelial (E) and Mesenchymal (M) Heterogeneity in Patient-Derived CTCs

**Figure 5:**
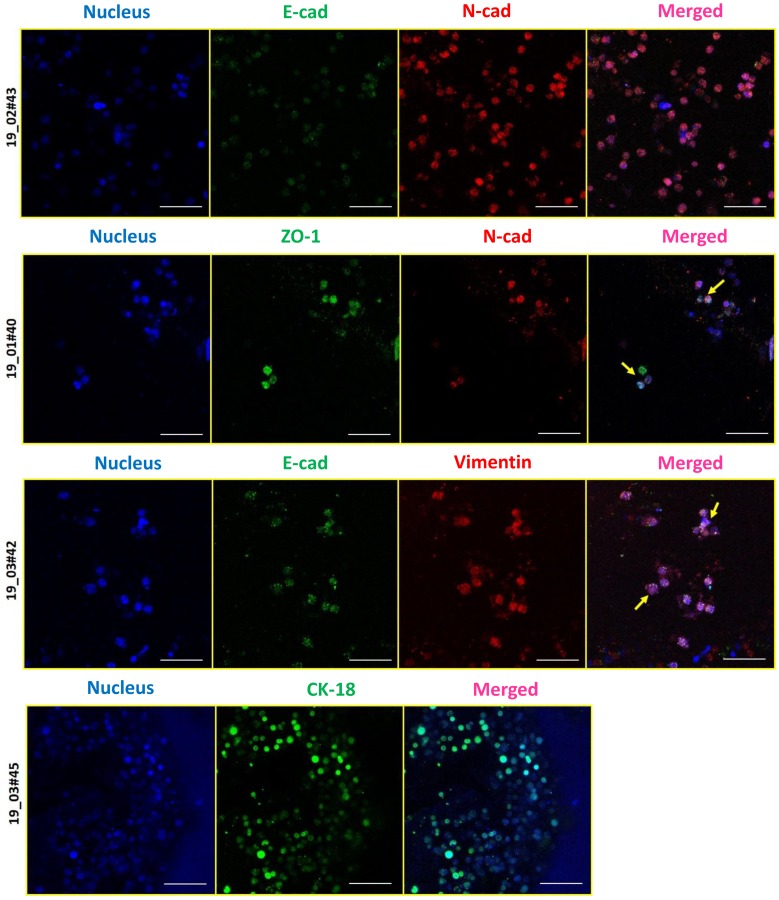
E-M characterization of CTCs derived from blood samples of breast cancer patients. Immunostaining revealed the presence of epithelial (E)-type CTCs expressing E-cad, ZO-1, or CK18 (green), and mesenchymal (M)-type CTCs expressing vimentin or N-cad (red). Cells were stained for nucleus (Hoechst 33342, blue). Imaging was performed using confocal microscope and maximum intensity projections are shown. Scale bar represents 40 μm. Images are representative of 4 independent patient samples.

Since our CTC culture strategy did not involve a prior enrichment with epithelial markers such as EpCAM, we exploited this system to investigate whether CTC subsets enriched by growing in 3D PCL scaffold showed the presence of epithelial (E) and mesenchymal (M)-type cells. For this, we analyzed the expression of sets of epithelial and mesenchymal markers. We selected a series of epithelial (panCK/CK18/ZO-1/E-cad) and mesenchymal marker (N-cad/Vimentin) markers which were first confirmed across breast cancer cell lines (Supplementary Figure S3A,B). Immunostaining of CTCs cultured in PCL scaffolds for the aforementioned markers revealed the presence of differential expression of both E- and M-type markers within the same patient, as well as across different patients (Figure 5). The result showed dual expression of both epithelial and mesenchymal markers suggesting the existence of intermediate EMT phenotype (Figure 5). Notably, M-type marker expression was more in most of the patient samples, suggestive of an intermediate mesenchymal phenotype, which well correlated with our earlier observation where we saw intermediate mesenchymal scores for the breast CTCs [19].

**Figure 6:**
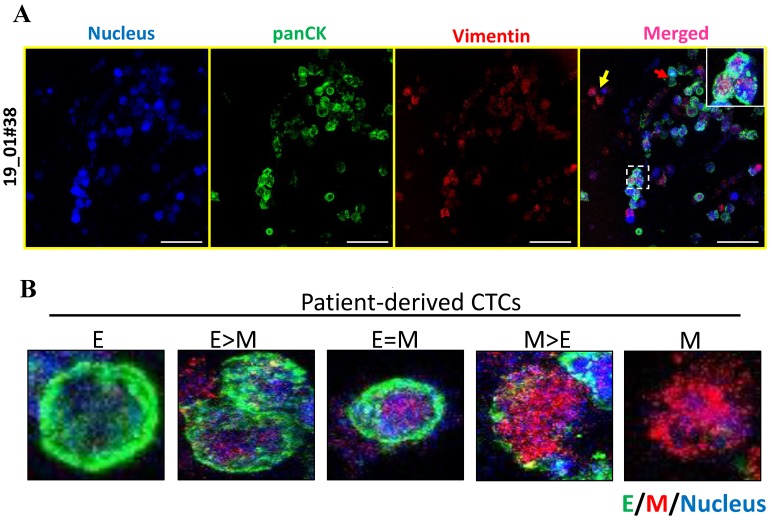
E-M heterogeneity and intermediate phenotypes in breast cancer patient-derived CTCs. (**A**) Co-staining for panCK and vimentin in patient-derived CTCs revealed the presence of marked heterogeneity with respect to E and M marker expression. (**B**) Representative fluorescence images of 5 different types of CTCs: E (exclusively), E > M, E = M, M > E and M (exclusively). Cells were counterstained for nucleus (Hoechst 33342, blue), E-type markers (green), and M-type markers (red). Imaging was performed using confocal microscope and maximum intensity projections are shown. Scale bar represents 40 μm (**A**). Images are representative of 2 independent patient samples.

To further address E-M heterogeneity, we first confirmed the dual expression of a combination of E (pan-CK)- and M (vimentin)-type markers across breast cancer cell lines MCF 7 and MDA MB 231 (Supplementary Figure S4). Dual immunofluorescence of panCK and vimentin on day 14 cultures of patient-derived CTCs showed the presence of heterogeneous expression of E and M markers (Figure 6A). A detailed analysis of the images revealed 5 categories of cells ranging from E (exclusively), E > M, E = M, M > E, M (exclusively) (Figure 6B) suggesting dynamic changes in epithelial and mesenchymal composition, similar to a recent report [20]. This dynamic E-M heterogeneity and the existence of intermediate/hybrid cells is supported by recent literature [21,22].

## 4. Discussion

CTCs are considered as surrogate markers for monitoring and evaluating patient treatment responses. Current advances in technology have enabled us to isolate these rare CTC populations. However, the real challenge lies in the successful expansion of these cells in culture conditions. Our group has previously established laser-ablated microwells-based method for the rapid expansion of CTCs [14]. Recently, we improvised the method using agar rather than tapered microwells to culture these cells [15]. However, 2D culture methods are incompetent to mimic natural structural organization. They also exhibit compromised morphology and differentiation. Also, deposition of the extracellular matrix is critical for proper attachment and proliferation. Several 3D scaffolds either use a material like agar, which fails to allow ECM deposition, or matrigel, which has an abundant ECM that might not mimic the environment faced by CTCs at the secondary site. To overcome these concerns, we developed a 3D culture method using 3D porous PCL scaffolds which allowed deposition of ECM from the cells derived from patient samples and enabled the culture of CTCs.

When RBC-depleted nucleated cell pellets from whole blood from breast cancer patient samples were cultured in the 3D porous PCL scaffolds, we observed cell clusters which were positive for F actin. Our SEM micrographs revealed the presence of ECM deposition on the PCL scaffolds. This observation was confirmed by immunostaining for Laminin protein, a component of the extracellular matrix. In this study, SEM micrographs have revealed the formation of inter-cellular connections and tumor like masses or tumoroids derived from nucleated patient-blood samples. CTCs are typically identified based on the expression of epithelial markers such as keratins, EpCAM and the absence of common leukocyte marker CD45 [23]. In accordance with this, we noted the presence of pan-cytokeratin positive and CD45 negative cells in our 3D cultures. We cannot rule out the possibility that these are epithelial tumor cells engulfed by fibroblasts and macrophages; further characterization needs to be done to rule this out. However, recent reports have suggested the existence of E-M heterogeneity based on the expression of both epithelial and mesenchymal markers [24]. We further characterized the epithelial and mesenchymal properties of CTCs in 3D PCL scaffolds. Our results indicated dual expression of both epithelial (pan CK) and mesenchymal markers (vimentin), revealing the presence of intermediate/hybrid EMT phenotype. Moreover, we also observed differential expression holds true for several other markers including E-cadherin, ZO-1, N cadherin and vimentin as well. Further investigation is required to validate the same with various histological subtypes of breast cancers (based on ER/PR/HER2 status).

The breast cancer patient-derived CTCs effectively reflect inter- and intra-patient heterogeneity in terms of E or M markers expression. Additionally, cell proliferation assays confirmed the expansion of viable nucleated cells in 3D culture system. Although our data showed proliferating cell population, and we have previously demonstrated the gradual depletion of other lineage cells [14], it still remains to be confirmed if CTCs are indeed proliferating in these scaffolds by undertaking a co-staining staining for Pan-CK, CD45, and Ki67. The proliferating cells as observed by Ki67 and BrdU positivity in our study could include both CTCs as well as other cell types, such as cancer associated fibroblasts and macrophages, which may provide a suitable microenvironment for the enrichment of CTCs. Nevertheless, our study revealed visualization of viable cytokeratin positive/CD45 negative cells, and the detection of ZO-1 alone and ZO-1/N-cad double positive cells in these scaffolds by the end of 14 days in culture. Thus, our study shows that culture of RBC-lysed, nucleated cells from patient blood in 3D-PCL scaffold can serve as a novel system for culturing CTCs. Taken together, our study has demonstrated an easy but effective 3D culture method for in vitro culture and possible expansion of CTCs. Hence, this could serve as an excellent platform for a deeper study of CTC biology and metastasis. Taken together, we conclude that our novel ex vivo 3D culture system would provide a more reliable system for future personalized drug-screening for cancer patients.

## Supplementary Materials

The following are available online at https://www.mdpi.com/2077-0383/8/9/1473/s1, Figure S1: 3D PCL scaffolds exhibit good porosity and pore inter-connectivity, and show the presence of cells, Figure S2: Active cell proliferation in breast cancer cell line (MDA-MB-231), Figure S3: Expression of epithelial (E) and mesenchymal (M) markers across breast cancer cell lines, Figure S4: E-M heterogeneity in breast cancer cell lines. Table S1: Clinical parameters of breast cancer patients (Invasive ductal carcinoma (IDC) analyzed in this study.
