# Supplementary material for: A Novel Ex Vivo System Using 3D Polymer Scaffold to Culture Circulating Tumor Cells from Breast Cancer Patients Exhibits Dynamic E-M Phenotypes
Source: J Clin Med. 2019 Sep 16;8(9):1473. doi: 10.3390/jcm8091473 (PMC6780381; doi:10.3390/jcm8091473)

Figure S1: 3D PCL scaffolds exhibit good porosity and pore inter-connectivity and show the presence of cells. (A) Representative SEM micrograph of scaffold in low magnification (i) and in higher magnification (arrows indicate porous structure of scaffold) (ii), single pore having a particular diameter of porogen used (250-425μm) (iii), arrows show pore interconnectivity (iv). Scale bar represents 5.0 mm in (i), 500 μm in (ii) and (iv), 400 μm in (iii). (B) Immunofluorescence images of F-actin (red) and nucleus (stained with Sytox green) in RBC-depleted nucleated cell pellet of patient samples cultured in 3D PCL scaffold for 14 days. Imaging was performed using Epi-fluorescence microscope. Scale bar represents 100 μm. Images are representative of 2 independent patient samples.

Figure S2 Active cell proliferation in breast cancer cell line (MDA-MB-231). (A) Immunostaining for Ki-67 protein showed active cell proliferation in MDA-MB-231 cells; top panel minus primary negative control, middle panel showing Ki67 positivity at 20X magnification (A) and bottom panel at 60X (B) magnification. Imaging was performed using Epi-fluorescence microscope, scale bar represents 100 μm (A, B).

Figure S3: Expression of epithelial (E) and mesenchymal (M) markers across breast cancer cell lines. Immunostaining indicates the differential expression of epithelial (E) type markers (pan-CK/CK18/E-cad/ZO-1) or mesenchymal (M) type markers (Vimentin/N-cad) in MCF7 and MDA MB 231 breast cancer cells (A and, B, respectively). Cells were stained for nucleus (blue), E-type markers (green), M-type markers (red). Imaging was performed using Epi-fluorescence microscope. Scale bar represents 100 μm.

Figure S4: E-M heterogeneity in breast cancer cell lines. (A) Dual staining for epithelial (panCK) and mesenchymal (vimentin) marker across two different breast cancer cell lines (MCF7 and MDA-MB-231).

Supplementary Table S1: Clinical parameters of breast cancer patients (Invasive ductal carcinoma (IDC)) analyzed in this study

Patient ID	Clinical status	Primary cancer	ER	PR	HER-2	Diagnostic stage	Age
18_09#31	LU, BO	Breast	-	+	+	IV	56
18_09#32	LI, BO	Breast	+	+	+	IV	52
18_09#33	CAN	Breast	+	+	-	IV	46
18_09#34	LU, BO	Breast	+	+	-	IV	34
18_09#35	LI, BO	Breast	+	+	+	IV	54
18_10#36	BO	Breast	+	+	+	IV	59
18_12#37	LU, LN	Breast	-	+	-	IV	26
19_01#38	LI	Breast	-	-	+	IV	53
19_01#39	CAN, LN	Breast	-	+	+	IV	37
19_01#40	LI	Breast	+	+	-	IV	49
19_02#41	BO	Breast	+	+	-	IV	42
19_02#42	LU	Breast	+	+	+	IV	60
19_02#43	LU, BO	Breast	-	+	+	IV	71
19_03#44	LU, BO	Breast	-	-	-	IV	73
19_03#45	LU, LI	Breast	+	+	-	IV	37
19_04#46	LU, BR	Breast	-	-	-	IV	44

Lung- LU, Liver-LI, Bone-BO, Brain- BR, Contralateral axillary node- CAN, Lymph node- LN

Figure S1.

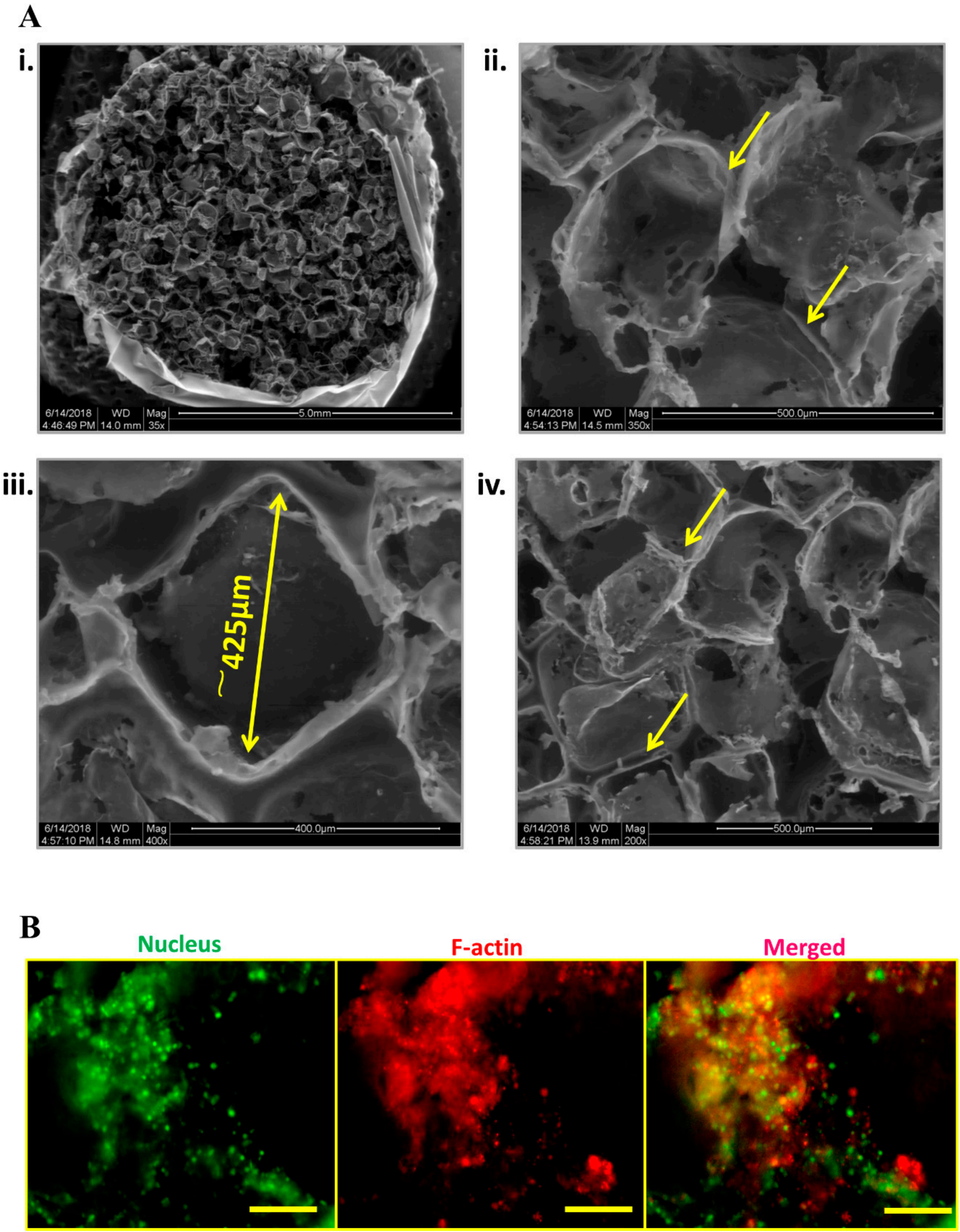
A
i.
ii.
iii.
iv.
~425μm
6/14/2018 4:46:49 PM WD 14.0 mm Mag 35x 5.0mm
6/14/2018 4:54:13 PM WD 14.5 mm Mag 350x 500.0μm
6/14/2018 4:57:10 PM WD 14.8 mm Mag 400x 400.0μm
6/14/2018 4:58:21 PM WD 13.9 mm Mag 200x 500.0μm
B
Nucleus
F-actin
Merged

Figure S2.

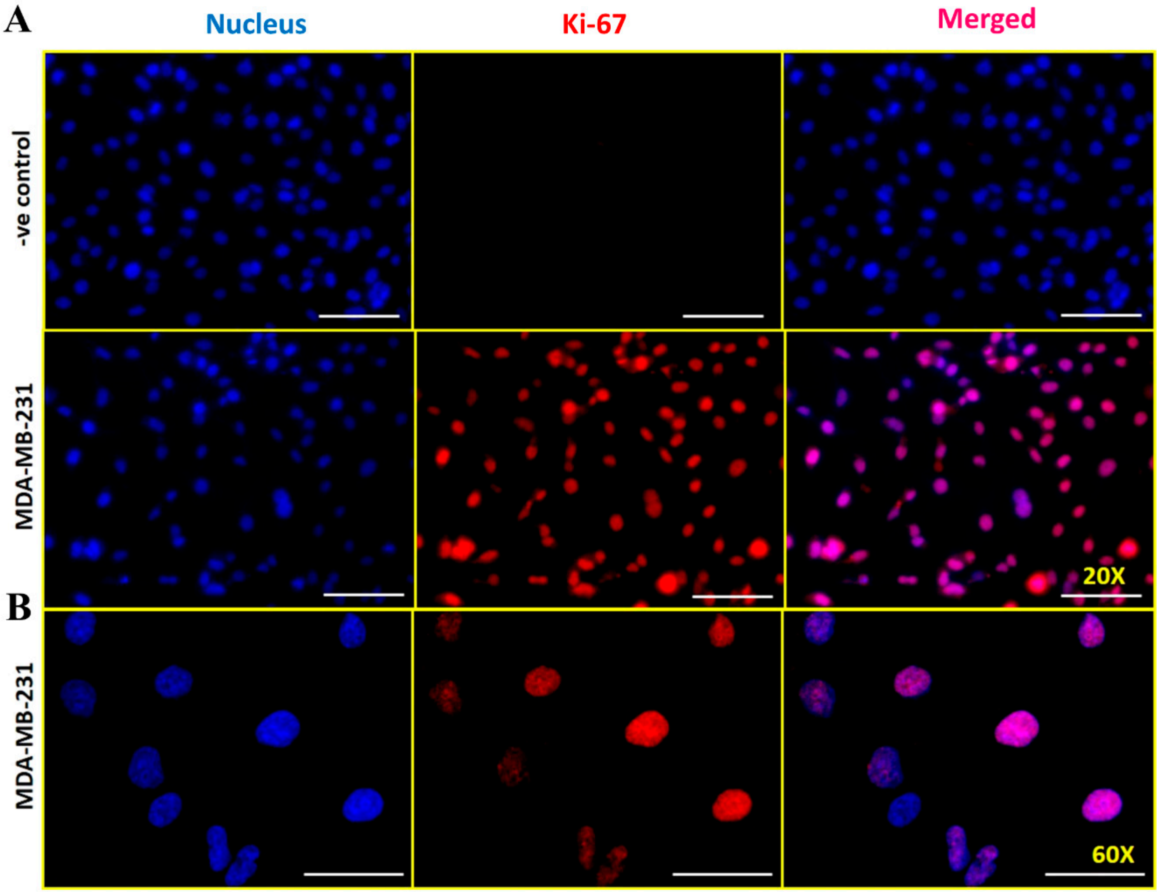

A
Nucleus
Ki-67
Merged
-ve control
MDA-MB-231
20X
B
MDA-MB-231
60X

Figure S3.

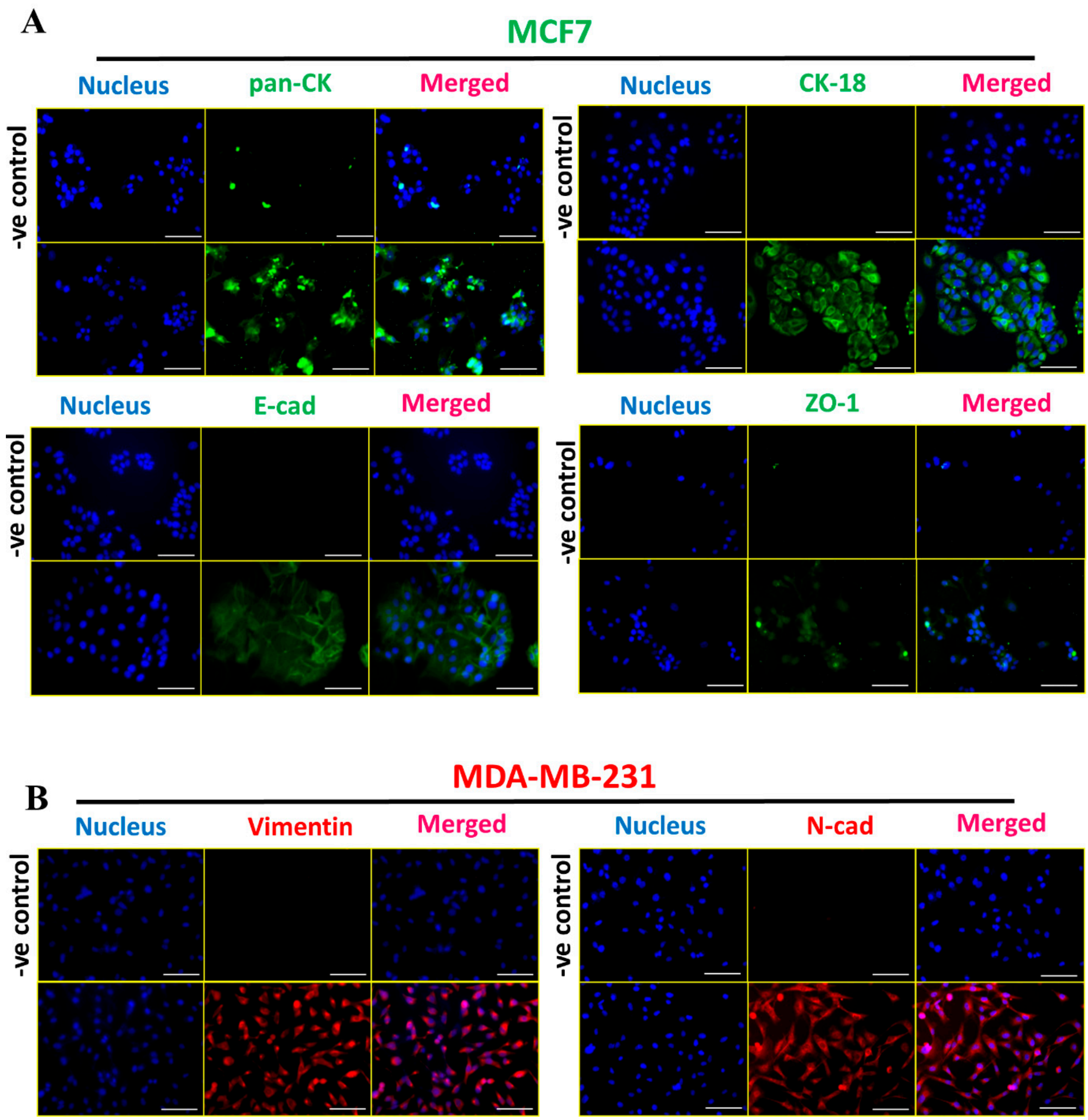
A
MCF7
Nucleus
pan-CK
Merged
Nucleus
CK-18
Merged
-ve control
-ve control
Nucleus
E-cad
Merged
Nucleus
ZO-1
Merged
-ve control
-ve control
B
MDA-MB-231
Nucleus
Vimentin
Merged
Nucleus
N-cad
Merged
-ve control
-ve control

Figure S4.

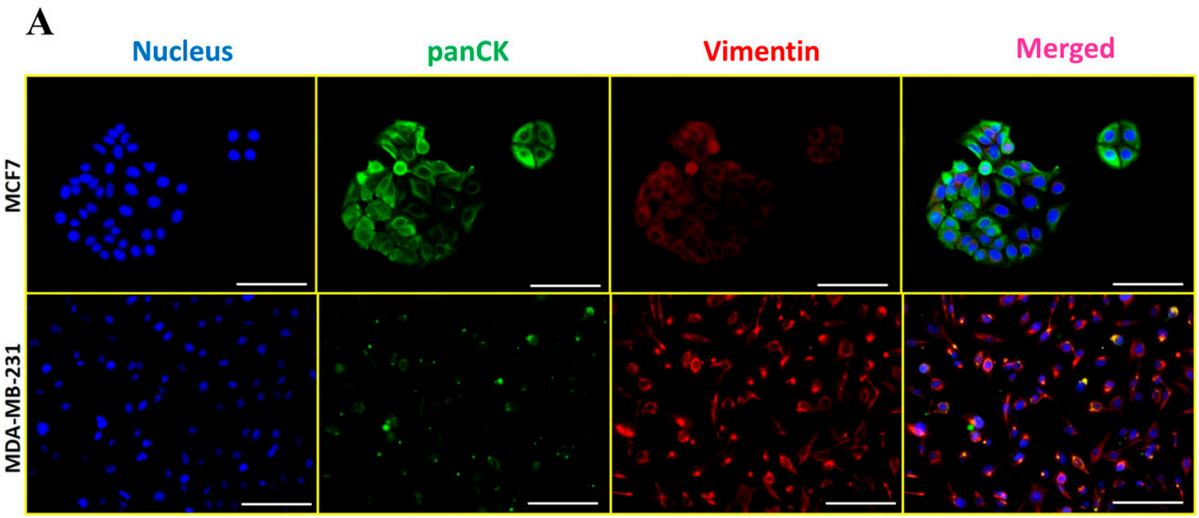

Supplement: Supplementary file 1 [file jcm-08-01473-s001.zip › jcm-561239-SI.pdf]
